# Hints on Dental Societies

**Published:** 1863-09

**Authors:** 


### Editorial.

### HINTS ON DENTAL SOCIETIES.

IN reading an editorial recently, we felt half inclined to offer a few "practical hints." We are told that "it is doubtless true that Dental Societies have done a vast deal of good for dentistry; but we believe they could be productive of still more good." Certainly, Mr. Editor; and that is the reason many dentists spend their time, money, and talents to keep them in existence. They are "productive of more good "than ever before," and and might do "still more good," if all who ought to, would give them their countenance and assistance; especially, if those who have given so much time and attention to the making of constitutions, by-laws, rules of order, etc.—who have helped to make so many good constitutions that they know exactly how to make more, and so many very bad ones, that they know how avoid them—if these would give dental societies their aid and counsel, they would "be productive of still more good." But the dental societies must lose all this, because they do not meet "the last week in August or first week in September," which we are told "would be much better" time for them to meet. And why better? Have Dentists more leisure then? Not in the West, certainly. Have they more mental energy? Possibly; but they do pretty well, even in July or August. We are sorry, both for the societies' sake and his own, that they do not meet at a time to suit the writer; but we fear the time suggested would be less suitable to most dentists than that now adopted.

After all his experience in good and bad constitutions, the editor has come to the conclusion that even a good one is worse than none. He says, "but we believe that a mere order of *conducting business* would better subserve the wants of associations than all the finely wrought principles of constitution and nicely balanced by-laws." We read of a man who, having lost his constition, was living on his *by-laws;* but this editorial physician prescribes still sparer diet for dental societies; and they will doubtless, thrive on it as rapidly as the wild ass fattens on the east wind. But then, it seems that even a defective constitution may do very well, if it is only rigidly adhered to—defects and all. We are thus told: "If a society adopts a constitution and by-laws and lets them alone, perhaps it would do very well; but nearly every new member who enters a society, thinks he must have the constitutions or by-laws altered if they do not meet his individualism." Our own experience with new members has been different. We have belonged to at least twenty societies, and not one new member in twenty has proposed such or any alteration of the constitution or by-laws. New members must be harder to please east than they are west.

But a society would perhaps do very well if it would let its constitution alone. And perhaps some have done very well who saw fit to alter their constitutions. The Mississippi Valley Association has changed its constitution more than once; and the American Association has made one change; and both are doing very well. A society does not deserve to do very well, nor even to exist, if it has so little energy that a new member can change its constitution and by-laws to "meet his individualism."

We are told, farther; "we believe the dental convention system is the best idea that has yet been hit on for dentists." Well, he ought to know, for he has been there himself. He has attended at least one whole meeting of the American Dental Convention, to say nothing of the fifteen minutes spent in the American Dental Association. But we believe in the convention too. It answers the end of its creation; and that can not be said of everything. The profession needs the convention, and must have it; but it needs local societies and a national delegated body as well. It needs both these means of progress, and they are not in the least incompatible, but, on the contrary, they mutually promote the interests of each other. The convention has greatly advanced the interest of the profession; but there are local societies in existence that are doing quite as much; and a leading argument in favor of the American Association is that it encourages the formation of local societies We can freely say that we know of no organization or institution in our profession whose prosperity we do not ardently desire. W.

